# The complete mitochondrial genome of the Pink dentex *Dentex gibbosus* (Perciformes: Sparidae)

**DOI:** 10.1080/23802359.2018.1467230

**Published:** 2018-04-26

**Authors:** Celestina Mascolo, Marina Ceruso, Giuseppe Palma, Aniello Anastasio, Tiziana Pepe, Paolo Sordino

**Affiliations:** a Department of Veterinary Medicine and Animal Production, University “Federico II”, Naples, Italy;; b Assoittica Italia, Rome, Italy;; c Biology and Evolution of Marine Organisms, Stazione Zoologica Anton Dohrn, Naples, Italy

**Keywords:** *Dentex gibbosus*, mitogenomics, Perciformes, Sparidae

## Abstract

The Pink dentex (*Dentex gibbosus,* Rafinesque 1810) is one of the most commercially important Sparidae species and it is often subjected to fraud. Here, we report the complete mitochondrial genome of *D. gibbosus*. The mitogenome is 16,771 bp in length and contained 13 protein-coding genes, 2 rRNA genes, 22 tRNA genes and 2 non-coding regions. The overall base composition of *D. gibbosus* mtDNA is: 27.8% for A, 28.60% for C, 16.5% for G, 27.05% for T.

The Pink dentex (*Dentex gibbosus*) is a commercially important Sparidae species inhabiting the West African coast from Portugal to Angola, including Madeira, Canary and Sao Tome-Principe archipelagos (Wirtz et al. 2008). This sparid species is also present in the Mediterranean Sea except for the north-western coast and the northern Adriatic Sea (Dooley et al. 1985). *Dentex gibbosus* is now classified as “Least Concern” in the Red List of Threatened Species in the Mediterranean Sea (Russell et al. 2014). Species substitution is very common in processed fishery products belonging to Sparidae family. In particular, *Dentex dentex* and *Pagrus pagrus*, are often fraudulently replaced with *D. gibbosus* (Katavic et al. 2000). Here, we describe the complete mitochondrial genome (mitogenome) of *D. gibbosus* (GenBank MG653593). A specimen caught in the Mediterranean Sea (N 38°25′15.1″ E 3°53′09.4″) was identified as *D. gibbosus* based on morphological features. DNA was extracted and is currently stored at Department of Veterinary Medicine and Animal Production, University “Federico II”, Naples, Italy. The mitogenome of *D. gibbosus* has been obtained from high-throughput sequencing on complete mitochondrial DNA with Illumina HiSeq 2500 System (Illumina, San Diego, CA). The complete sequence is 16,771 bp long, containing 13 protein-coding genes, 2 ribosomal RNA genes (12S rRNA and 16S rRNA), 22 transfer RNA genes (tRNA) and two non-coding regions (D-loop and L-origin). Mitochondrial arrangement and gene distribution are in agreement with the classic vertebrate mitogenomes (Wang et al. 2008). The majority of mitochondrial genes are encoded on the heavy strand, while the NADH dehydrogenase subunit 6 (*ND6*) and eight tRNA genes [Gln, Ala, Asn, Cys, Tyr, Ser(UCN), Glu, Pro] are encoded on the light strand. Base composition is similar to other Sparidae mitochondrial genomes, with 27.8% for A, 28.60% for C, 16.5% for G, and 27.05% for T (Ceruso et al. 2018). All the protein-coding genes started with an ATG start codon but *COI* and *ND4*, which started with GTG. Stop codons were of four types, i.e. TAA (*ND1*, *ATP8*, *ATP6*, *ND4L*, *ND5, ND6*), AGG (*COI*), T (*COII*, *ND3, ND4*, *CYTB*) and TA (*ND2*, *COIII*). The 12S and 16S rRNA genes were located between the *tRNA^Phe^* (GAA) and *tRNA^Leu^* (TAA) genes, and were separated by the tRNA^Val^ gene as in other vertebrates (Li et al. 2016). The 22 tRNA genes vary from 66 to 74 bp in length. The 1091 bp long control region is located between *tRNA^Pro^* (TGG) and *tRNA^Phe^* (GAA). The non-coding region (L-strand origin of replication) is 40 bp long and is located between *tRNA^Asn^* (GTT) and *tRNA^Cys^* (GCA). To validate the phylogenetic position of *D. gibbosus*, we construct a phylogenetic tree using MEGA6 software (Tamura et al. 2013) (Figure 1). The resultant phylogeny shows that *D. gibbosus* is closely related to *D. dentex*, in agreement with Chiba et al. (2009). Results of this study provided useful genetic information for further studies on phylogeny, species identification and population genetics in Sparidae species.

**Figure 1.**
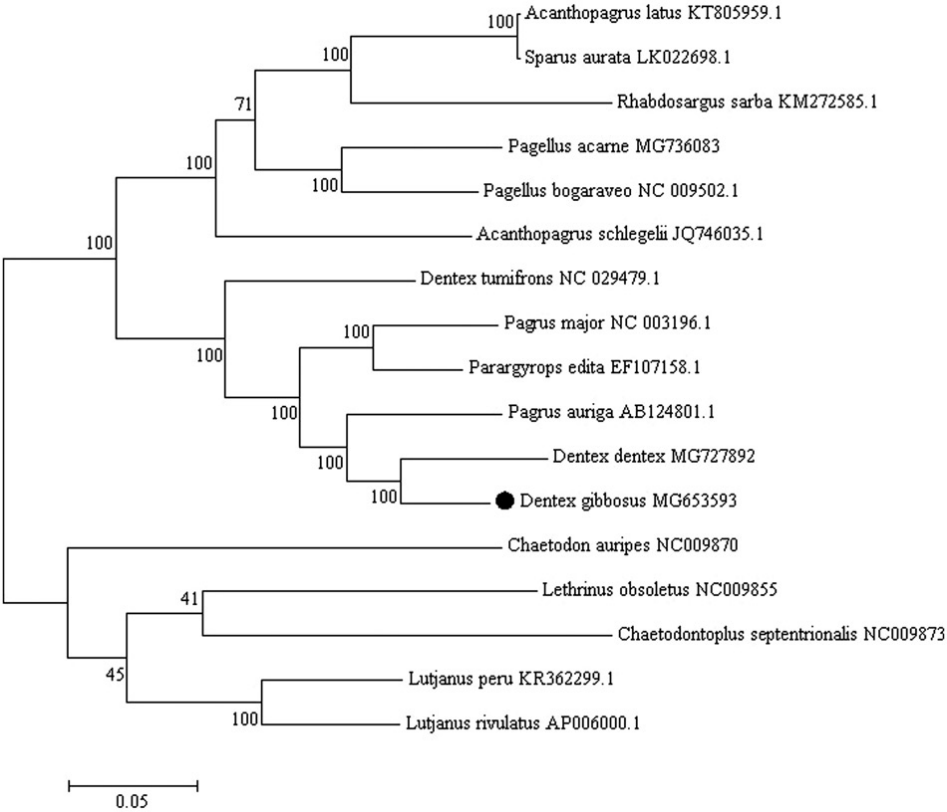
Phylogenetic analysis of *D. gibbosus* based on the entire mtDNA genome sequences of 11 sparid fishes available in GenBank. Five outgroup species (*Lutjanus peru*, *Lutjanus rivulatus*, *Lethrinus obsoletus*, *Chaetodontoplus septentrionalis* and *Chaetodon auripes*) were selected and the maximum likelihood method was used. Numbers above the nodes indicate 1000 bootstrap values. Accession numbers are shown behind species names.
